# The Importance of an Active Case Detection (ACD) Programme for Malaria among Migrants from Malaria Endemic Countries: The Greek Experience in a Receptive and Vulnerable Area

**DOI:** 10.3390/ijerph17114080

**Published:** 2020-06-08

**Authors:** Maria Tseroni, Maria Georgitsou, Agoritsa Baka, Ourania Pinaka, Danai Pervanidou, Maria Tsironi, Panagiota Bleta, Maria Charvalakou, Ioanna Psinaki, Martha Dionysopoulou, Antonia Legaki, Annita Vakali, Elina Patsoula, Evdokia Vassalou, Spyridoula Bellou, Vasilis Diamantopoulos, Theano Georgakopoulou, Varvara Mouchtouri, Sotirios Tsiodras, Nicos Middleton, Andreas Charalambous, Vasilios Raftopoulos, Christos Hadjichristodoulou

**Affiliations:** 1 National Public Health Organization of Greece, 3-5 Agrafon St., 15123 Marousi, Greece; a.baka@eody.gov.gr (A.B.); d.pervanidou@eody.gov.gr (D.P.); a.vakali@eody.gov.gr (A.V.); t.georgakopoulou@eody.gov.gr (T.G.); tsiodras@med.uoa.gr (S.T.); vraftop1@gmail.com (V.R.); 2 Department of Nursing, Cyprus University of Technology, 30 Arch. Kyprianos Str., 3036 Limassol, Cyprus; nicos.middleton@cut.ac.cy (N.M.); andreas.charalambous@cut.ac.cy (A.C.); 3 Department of Hygiene and Epidemiology, Faculty of Medicine, University of Thessaly, 22 Papakyriazi St., 41222 Larissa, Greece; marma8920@gmail.com (M.G.); rpinaka@gmail.com (O.P.); pennympleta@hotmail.com (P.B.); marie_87@hotmail.com (M.C.); psinaki.iwanna@gmail.com (I.P.); mouchtourib@med.uth.gr (V.M.); xhatzi@med.uth.gr (C.H.); 4 Department of Nursing, University of Peloponnese, Karaiskaki 70, 22100 Tripoli, Greece; mtsironi@otenet.gr; 5 General Hospital of Sparti, 23100 Lakonia, Greece; dlab@hospspa.gr; 6 General Hospital of Molaoi, 23052 Lakonia, Greece; mikroviologiko@hosmol.gr; 7 Public Health Parasitology and Entomology, Department of Public Health Policy, School of Public Health, University of West Attica, 196 Alexandras Avenue, 11521 Athens, Greece; epatsoula@uniwa.gr (E.P.); evassalou@uniwa.gr (E.V.); 8 Directorate of Public Health, Prefecture of Peloponnese, 34 El. Venizelos, 22132 Tripoli, Greece; mpellous@yahoo.gr (S.B.); diamantopoulosv@yahoo.com (V.D.); 9 Medical School, University of Athens, 75 Mikras Asias, 11527, Athens, Greece

**Keywords:** malaria, plasmodium vivax, active case detection, migrants, Greece

## Abstract

Greece has been malaria-free since 1974. In October 2011, following an outbreak of 36 locally acquired malaria (LAM) cases in Evrotas Municipality, a Pro-Active Case Detection (PACD) program for malaria was implemented among migrants from malaria-endemic countries, to support early diagnosis and treatment of cases. We evaluated the PACD program for the years 2012–2017 using indicators such as the number of locally acquired cases, the detection rate/sensitivity and the timeliness of diagnosis and treatment. We visited each migrant home every 7–15 days to screen migrants for malaria symptoms, performing Rapid Diagnostic Tests (RDTs) and blood smears on symptomatic patients. We estimated: (i) the number of malaria cases detected by the PACD, divided by the total number of reported malaria cases during the same period among the same population; (ii) the time between onset of symptoms, diagnosis and initiation of treatment. The total number of migrants who were screened for malaria symptoms for the years 2012–2017 was 5057 with 84,169 fever screenings conducted, while 2288 RDTs and 1736 blood smears were performed. During the same period, 53 imported *P. vivax* malaria cases were detected, while incidence of malaria among migrants was estimated at 1.8% annually. Ten and one LAM cases were also reported in 2012 and 2015, respectively. Sensitivity of PACD ranged from 86% to 100%; median timeliness between onset of symptoms and diagnosis decreased from 72 h in 2012 to 12 h in 2017 (83% decrease), while timeliness between diagnosis and treatment initiation was 0 h. The implementation of PACD could be considered an effective prevention and response tool against malaria re-introduction.

## 1. Introduction

Malaria remains the most prevalent vector borne disease worldwide. According to the World Health Organization’s (WHO) latest estimates, 228 million cases occurred globally in 2018, resulting in 405,000 deaths. Most cases (93%) were reported from the WHO African region, followed by the WHO South-East Asian Region with 3.4% of cases and the WHO Eastern Mediterranean Region with 2.1% [1].

*Plasmodium falciparum* is responsible for most of the severe disease and deaths due to malaria and prevails in sub-Saharan Africa, while *Plasmodium vivax* generally causes less severe disease but has the widest geographical distribution of all species, occurring most frequently in Southeast Asia and some areas of South America [1].

In this context, the WHO global strategy for malaria (2016–2030) aims to reduce malaria case incidence and mortality rates by at least 90%, eliminate malaria in at least 35 countries, and prevent a resurgence of malaria in all countries that are currently malaria-free. In order to achieve these goals the strategy focuses on three pillars: (i) ensuring universal access to malaria prevention, diagnosis and treatment, (ii) strengthening efforts towards elimination and maintaining a malaria-free status, (iii) transforming malaria surveillance into a core intervention using innovation, research and strengthening of the enabling environment as supporting elements. In order to attain elimination and prevent re-establishment, the role of Active Case Detection is crucial as it consists of screening for fever at both community and household levels, sometimes in population groups that are considered high risk [2].

Within the WHO European region, malaria cases are mostly imported by international travelers and immigrants. The proportion of imported malaria cases due to immigrants has increased over the last decade; higher rates are associated with settled immigrants who travel to visit friends and relatives (VFRs) [3,4,5]. According to the European Centre for Disease Prevention and Control (ECDC), the number of confirmed malaria cases reported in the EU/EEA for the year 2017 was 8401 [6]. In addition, 21 confirmed cases were reported as acquired in the EU: seven by both Greece and Italy, three by the UK, two by France and one each by Germany and Spain [7].

Malaria was endemic in Greece until the 1960s. In 1974 the country became malaria-free. Since 2009, introduced *Plasmodium vivax* cases have been reported on an almost annual basis [8,9,10,11].

In 2011, a cluster of 36 *Plasmodium vivax* locally acquired malaria cases occurred in a specific, small geographical area of the Evrotas Municipality in the Peloponnese region of southern Greece. The Evrotas area was one of the historical hot spots of malaria prior to the elimination of the disease [10,12].

After the outbreak in 2011 and following experts’ advice from the WHO and ECDC to actively search for malaria cases in the area, the National Public Health Organization (NPHO) of Greece/formerly the Hellenic Center for Disease Control and Prevention (HCDCP), designed and implemented a malaria Active Case Detection (ACD) program to identify malaria cases in a timely manner at the community level among symptomatic migrant farm workers. The aim was to interrupt local malaria transmission, and to prevent malaria re-introduction and re-establishment in the area [10,13,14,15].

In addition, since 2012 a number of other response interventions were implemented including intensified vector control activities, such as Indoor Residual Spraying (IRS) and distribution and use of Long Lasting Insecticide-treated Nets (LLINs) [10,16].

In parallel with the ACD, targeted Mass Drug Administration with anti-malarials for *P. vivax* was administered in 2013 and 2014 to all migrants from endemic countries residing in the specific area. This was done from the beginning of the mosquito season as an additional measure to eliminate the parasites. In 2015, the same activity was implemented after the detection of an introduced case in September [11,16,17,18].

Between 2012–2014 all activities were supported by the Malaria—West Nile Virus (MALWEST) project funded by the Ministry of Health and the European Commission [19].

The aim of this study is to describe the ACD program implemented during 2012–2017 in the receptive and vulnerable Evrotas area in Greece, which is a developed malaria free country, to present the results including the achievement of surveillance indicators, and discuss the challenges in its implementation.

## 2. Methods

This is a retrospective research study that has been conducted in a sample of migrants from malaria endemic countries residing in the receptive and vulnerable Evrotas area in Greece. An ACD malaria program was implemented under the supervision and coordination of several public health authorities (NPHO, Medical School University of Thessaly, Public Health Directorate of Peloponnese Region).

The ACD program included both Re-Active Case Detection (RACD) and Pro-Active Case Detection (PACD). RACD was defined as the screening of local and migrant populations around a confirmed case. PACD was defined as screening with regular visits to households of migrants from malaria endemic countries for symptoms compatible with malaria.

### 2.1. Study Area and Population

The particular geographic study area which was previously a malaria hot spot prior to malaria elimination in Greece comprises a series of irrigation canals, the delta of the Evrotas River, coastal wetlands and one lake with brackish water, an ecosystem which favors the presence of malaria vectors. The surrounding area is primarily large scale agricultural land consisting mainly of orange and olive groves, which are the main source of income for most residents. Previous entomological studies showed the presence of *Anopheles sacharovi*, a known vector for the *Plasmodium vivax* parasite in the area, as well as in other parts of the country [9,12,20,21]. Additionally, the area hosts a large community of migrant farm workers originating mostly from the Indian subcontinent where *P. vivax* is endemic, increasing the possibility of parasite importation. Most of the migrants work seasonally and are undocumented, which limits their access to health care services. This migrant population tends to move frequently within Greece for seasonal agricultural work [12].

The study population includes all the migrants from malaria endemic countries residing in the area and covered by the PACD program, which fluctuated according to labor needs as well as migrant flows into Greece.

The PACD program was implemented annually between 2012–2017 starting in April or May until the end of November, according to the estimated possible “transmission period” of malaria parasites, which depends on environmental factors (temperature, rainfall), the circulation of *Anopheles* mosquitoes and the fluctuation of the migrant population, as well as the available public health resources. In 2012, the program was conducted throughout the year in response to the 2011 cluster. Moreover, in 2015 the program began on 31 July due to a lack of financial resources that was related to the economic crisis in the country.

**Figure 1:**
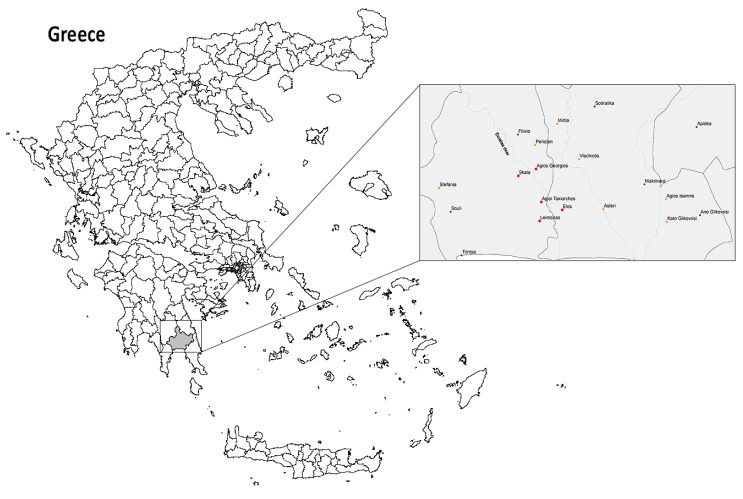
Malaria Pro-Active Case Detection program implementation in 12 villages of Evrotas Municipality (gray colored on the map of Greece): five villages at the epicenter of the 2011 outbreak (red colored bullets) and seven surrounding villages (orange colored bullets).

The PACD program was implemented in 12 villages (totaling 137.313 sq km and approximately 10,000 Greek residents) [22] comprised of five villages at the epicenter of the 2011 outbreak (red colored bullets in Figure 1) and seven surrounding villages (orange colored bullets in Figure 1) with significant migrant farm worker populations.

### 2.2. Description of the Surveillance Program

The main objective of the PACD program was the early detection of malaria; therefore, adherence to a regular visitation schedule of migrant households was required. The frequency of the visits was every 7–15 days, with the aim of weekly visits for the peak season (July–October) each year.

The field team mapped and coded migrant households in each village by using a GPS device (GARMIN NUVI42 Essential Series) with the coordinates entered in ArcGIS V.10.1 GIS software (ESRI; Redlands, CA, USA). A sticker with the household code number was located in a prominent place in each household, which included advice for communicating with the field team through a hotline number in case of malaria symptoms.

Field teams, each consisting of a health professional and a mediator performed ACD. An on call medical doctor supported the field team and provided medical services when there was a need.

The field team screened for fever and other malaria compatible symptoms, and tested every suspected malaria case. A “suspected malaria case” was defined as any person with documented temperature ≥37.0 °C, or history/complaints of fever and/or other malaria-compatible symptoms (headache, fatigue, myalgia) in the previous 7–15 days [23,24].

#### 2.2.1. Data Collection Tools

A number of data collection tools were developed for the needs of the PACD program, which included: (1) migrant household form (type of household, number of rooms, electricity, running water, heating, possible mosquito breeding sites in the vicinity, mosquito protection practices such as window nets, use of fan or air conditioning), (2) migrant registration form (age, sex, years of education, history of malaria or recent travel to country of origin, history of tuberculosis and hepatitis disease, time since arrival in Greece and in Evrotas, documentation), (3) migrant household visit form for each visit (including possible migrant movement from the household, temperature and symptoms per resident, performance), (4) malaria testing form (demographics, symptoms and date of onset, time since arrival in Greece, malaria history, RDT performance and results, blood sampling and results (from microscopy, PCR)), (5) malaria treatment form (demographics, date of diagnosis, Plasmodium species, daily directly observed treatment schedule and possible side-effects).

Data was entered daily into an Epi Info database (version 3.5.4, CDC, Atlanta, GA, USA) which was developed specifically for the needs of the PACD program. The NPHO is authorized by law to maintain the data base.

#### 2.2.2. Registration Process

After informed consent was obtained, every migrant resident in the study area was initially registered using the migrant registration form and informed about malaria symptoms, prevention measures and treatment, as well as the role of the program in the area. Information materials in the appropriate language and the 24/7 hotline number were distributed. A unique code was assigned to each migrant, and the team developed a specific procedure to avoid duplication due to mobility of the population. Therefore, each migrant was registered once during the study period even if they moved several times in and out of the study area. To identify new migrants in the study area, the snowballing technique was repeatedly used, i.e., by asking every migrant if he knew other migrants that had recently arrived.

#### 2.2.3. Site Visits

During each scheduled household visit, the following algorithm of actions was applied:

(i) New migrants in the area were registered and all residents were screened for malaria compatible symptoms. Axillary temperature was measured and recorded in the household visit form.

(ii) For suspected malaria cases the field team performed an RDT for malaria and/or blood sampling according to the protocol, and completed the malaria testing form. The RDTs used met the WHO’s recommended selection and procurement criteria (with a *P. vivax* panel detection score of 91.4% at 200 parasites/μL) [25,26].

(iii) In the case of a negative RDT for malaria in a person reporting compatible symptoms without another obvious cause of infection, an RDT and blood smear for microscopy were repeated at least two more times, approximately every 12–24 h before ruling out the diagnosis of malaria.

(iv) In the event of a positive RDT for malaria, the migrant was examined by a physician in the nearest health facility (a primary health care center) to clinically evaluate the severity of disease and the possible need for hospital admission. A complete blood count was also examined to verify anemia and thrombocytopenia. In case of mild clinical symptoms, good clinical condition and mildly affected blood test results, the patient was treated and further monitored by the field team in their household. As all introduced and imported malaria cases in Greece in this context are caused by *P. vivax*, Directly Observed Therapy (DOT) with chloroquine and primaquine was administered. Chloroquine was initiated immediately and primaquine was also administered as DOT, after G6PD testing, for 14 days. The malaria treatment form was completed at the daily follow up for general condition and possible adverse events.

Laboratory confirmation of the parasite by classical microscopy was conducted at the local hospitals and the Malaria Reference Laboratory, where PCR was additionally used.

(v) Each malaria case was reported to the NPHO of Greece and an epidemiological investigation was conducted to classify it as imported or locally acquired using a structured questionnaire. In addition, a reactive case detection (focus investigation) was performed by the field team, according to the operational protocol.

### 2.3. Field Teams Tasks

A minimum of two field teams were deployed on a daily basis, while a third team assisted either with fever screening or data entry. Moreover, a collaborating physician provided primary health care services and medical advice to migrants, e.g., consultations on upper respiratory and skin infections, and musculoskeletal complaints.

While each year the needs of the program varied slightly, the total number of field staff used for PACD activities included four health professionals (4 × 140 person-days per year), four mediators (4 × 120 person-days per year) and two drivers (2 × 140 person-days per year). Additionally, the program required one senior health professional as field coordinator (120 person-days per year), administrative support (one individual × 60 person-days per year), informatics support (one individual × 20 person-days per year) and a part-time medical professional/physician for primary health care services (20 person-days per year).

### 2.4. Definitions

A malaria case was classified by epidemiological criteria as imported if the infection was acquired outside Greece, and as locally acquired if the case was contracted locally. *P. vivax* cases among migrants were classified as imported if travel from an endemic country to Greece took place within three years before symptom onset [15,23]. For some migrants who declared more than three years (e.g., four years) of entry into the country, cases were also considered as imported since this was self-declared and not documented, and many migrants travelled back to their countries and returned to Greece during this period.

A “suspected malaria case” was defined as any person with documented temperature ≥37.0 °C, or history/complaints of fever and/or other malaria-compatible symptoms (headache, fatigue, myalgia) in the previous 15 days.

For the purpose of this analysis we defined as febrile cases individuals with documented temperature at the time of screening ≥37.5 °C, as low grade fever cases persons with documented temperature at the time of screening 37 °C–37.4 °C, and as afebrile cases persons with documented temperature at the time of screening <37 °C [24].

### 2.5. Surveillance Indicators

The PACD program was evaluated according to the following indicators, adapted from the WHO guidance on Malaria Elimination [14,15]:

- Number/proportion of locally acquired malaria cases exposed in Evrotas
- Sensitivity: Number/proportion of imported malaria cases detected through PACD
- Median time from onset of symptoms (fever) to first contact with the health system (target 24–48 h)
- Median time from first contact with the health system to malaria testing (target < 12 h)
- Median time from onset of symptoms (fever) to diagnosis (target: 24–48 h)
- Median time from diagnosis to treatment onset (target < 12 h)

### 2.6. Data Analysis

All migrants from malaria endemic countries recorded in the Epi Info database from 2012 to 2017 were included in the analysis. Descriptive analysis was conducted by using EPI Info software (version 3.5.4, CDC, Atlanta, GA, USA) and the statistical package SPSS 21.0 (IBM SPSS Inc., Armonk, NY, USA).

Patients were divided into four subgroups according to the year of their illness (2012, 2015, 2016 and 2017). Data were checked for deviation from normal distribution (Shapiro-Wilk normality test). Kruskal-Wallis tests were performed for continuous data. Statistically significant difference was considered when *p* value was <0.05.

As the total number of migrants screened during the weekly or bimonthly visits varied from one visiting period to another due to constant movements of this mobile population, the median number of migrants who were screened during the visiting periods was estimated, as well as the median number of households visited per year.

The indicator labeled “sensitivity” under the PACD program was calculated by estimating the number of malaria cases among migrants from endemic countries detected by the PACD program, divided by the total number of reported malaria cases during the same period and in the same population of the study area.

Incidence was calculated as the number of new malaria cases in the migrant population under surveillance. Due to high mobility of the particular population of migrant workers, the denominator used was the median number of migrants screened during the annual seasonal operation of the program.

Time to event data was analyzed using Kaplan–Meier statistics. Breslow (Generalized Wilcoxon) tests (overall and pairwise comparisons) were used to compare the Kaplan–Meier curves of patients per year. We used Breslow because it tests equality of survival functions by weighting all time points by the number of cases at risk at each time point.

## 3. Results

The demographic characteristics of migrants are summarized in Table 1. During 2012–2017, the total number of migrants registered in the PACD database was 5057. The majority of the migrants screened were of Pakistani nationality, originating from the province of Punjab. The median age of migrants in 2012–2017 was 24 (9–65) years, and all but one were men.

The median time period from their arrival in Greece to the day of their first contact with the field team and their registration in the database was much higher for the years 2012–2014 compared with the years 2015–2017 (Table 1).

The percentage of migrants who reported known malaria history in their country of origin was 3.6% (184/5057). Among them 6% contracted malaria.

There was a variance in the median number of migrants who were screened for malaria symptoms during the weekly or bimonthly visits (Table 2). The number of fever screenings per annual season varied accordingly by the number of migrants, the frequency of visits (7–15 days) and the deployment of the program each year.

From 2012 to 2017, a total of 53 *P. vivax* malaria cases among migrants from endemic countries was reported. In 2013 and 2014, no malaria cases were reported from the particular area (Table 3).

Taking into consideration the periods of operation of the malaria PACD program every year, the calculated sensitivity of the program ranged from 86% to 100%. A limited number of malaria cases were detected passively as they sought medical care directly through the local health care services (Table 4).

Table 5 summarizes the main demographic and clinical characteristics of the malaria cases among migrants from endemic countries in Evrotas between 2012–2017. Although fever is described as one of the main presenting symptoms for the disease, the majority of our cases presented with low grade fever. Another important finding was the relatively short interval between onset of malaria symptoms and arrival in Greece, with a median time interval ranging from 30 days in 2015 to 210 days in 2012.

Regarding the remaining surveillance indicators (Table 6), the median time from first symptom onset to diagnosis was gradually reduced from three days in 2012 to half a day in 2017. According to Table 7, there was a statistically significant difference in the number of days (from the onset of symptoms to diagnosis) between 2012–2017 and 2016–2017.

Furthermore, the program ensured that anti-malarial treatment was administered immediately after a positive RDT.

## 4. Discussion

This paper presents the implementation of a malaria Pro-Active Case Detection (PACD) program in a developed country, which recorded a limited number of sporadic introduced cases and a steadily increasing annual number of imported *P. vivax* malaria cases in 2013–2017. Introduced malaria cases have been detected in many European countries in recent years [3,4,5,27,28]. Since the competent vector (*Anopheles sacharovi*) is present in Greece, there is always a risk of malaria re-introduction in high risk receptive areas. Thus, based on our results the implementation of a PACD program could be considered an effective prevention and response tool against malaria re-introduction.

Our study population originated mainly from Pakistan, where *P. vivax* prevalence ranges from 2.4% in Punjab province to 10.8% in Sindh province [29]. Taking into consideration that underreporting is very likely, we assume that a significant percentage of the Pakistani migrants have been exposed to *P. vivax* since childhood and are carriers of *P. vivax* hypnozoites, while their relapses can be asymptomatic or minimally symptomatic as is indicated from our results and other related studies [30,31,32,33,34]. Detection of hypnozoites through a biological method is currently not possible, and serology is not widely accepted as a potential diagnostic method of infection, while it is difficult to implement systematically [35,36]. In addition to the above, further barriers due to documentation status, low risk perception of malaria and language barriers discourage these migrants from accessing health care services promptly. Therefore, passive surveillance and Reactive Case Detection are not enough to guarantee prevention of re-introduction episodes in a vulnerable and receptive area, as proven by the 2011–2012 outbreak in Evrotas, Peloponnese. Additional strategies implementing pro-active surveillance are needed to promptly detect new malaria cases, which can pose an infection risk to the local anopheles mosquito population [37,38]. ACD malaria programs are recommended by the WHO for countries in the elimination and prevention of re-introduction phases of malaria control, mainly in defined populations (“hot-pops”) living permanently in a certain area (“hotspots”) [15,37,38].

Our PACD program has special characteristics since it was conducted in a developed country targeting a hard-to-reach population of undocumented migrant workers with high mobility. According to Sturrock et al. [38], a mobile population constitutes a limitation for the success of a PACD program; however, we developed several procedures to overcome the relevant challenges. We designed processes for locating, registration, verification and follow up for each migrant staying in the area for more than 24 h, leading to the development of our ACD databases and procedures.

In 2013–2014, the national migration policy enforced strict measures against non-documented entry into Greece, i.e., closure of the land borders with Turkey at Evros and detention for any undocumented migrant arrested [39]. On the contrary, in 2015 very high migration flows were reported through the islands of the East Aegean, with about 1,000,000 migrants moving through Greece towards Central and Northern Europe [37]. The increased migration flows also presented an opportunity for the farm worker population to migrate from Greece to other European countries. Since then we have noted a marked decrease in their median stay in Greece before arriving at Evrotas, to approximately one month.

In 2016 and 2017, the migration flows at a national level had decreased [40]; however, the number of newly arriving migrants in Evrotas had actually increased, and their median age had decreased from 26 to 22 years of age.

Being undocumented usually makes migrants hesitant in their approach to health care services, and in their relationship with the PACD program which is perceived as a governmental activity, especially upon first contact. A Knowledge Attitudes and Practices (KAP) study conducted in 2013 in the same migrant population from malaria endemic countries residing in Evrotas showed that the level of malaria awareness among migrants was suboptimal, access to the main local healthcare facilities was limited, and poor housing conditions limited effective mosquito protection [41]. Consequently, efficient communication of health messages was a challenge. In addition, due to their low risk perception of malaria and their oftentimes mild relapse symptoms, it was difficult to persuade the study population to immediately notify the ACD team when they felt ill. The severity of malaria as an urgent public health issue in Greece was not easily understood and the rapid turnover of the population necessitated the constant repetition of health messages and advice. Mediators are instrumental in alleviating concerns and improving patient understanding and compliance with guidance and/or treatment.

*P. vivax* cases were usually detected in migrants with a limited reported stay in Greece, given that no locally acquired cases among natives were reported in the same period from the same area. The exception was the 2012 season, when a number of cases in migrants residing in the area of Evrotas may have been locally acquired (even indigenous), as indicated afterwards by recent molecular genotyping studies [42,43].

Approximately one in four malaria cases reported a history of malaria in their country of origin. A 1.8% incidence of imported *P. vivax* malaria was calculated annually in the area, which was similar to an incidence of around 2% in a Swedish study conducted in asylum seekers from Eritrea [44].

Regarding the cases detected by the program, less than 50% of migrants had a documented temperature of ≥37.5 °C at the time of diagnosis, inferring to a mild clinical presentation, while approximately 30% of all reported cases had no documented fever (T < 37 °C). The mild clinical picture also facilitated the outpatient management of the vast majority of our cases by the program staff, under a Directly Observed Therapy (DOT) protocol including primaquine eradication treatment. As hospitalization is a source of anxiety for the migrants due to a lack of mediators in the hospital, their lack of documentation and a lack of interaction with their relatives, migrants welcomed treatment at home. Only one case per year required admission, due to severe thrombocytopenia.

During the implementation of the PACD program, the surveillance indicators improved significantly over the years as no locally acquired malaria cases were reported in 2016–2017, even though the number of newly incoming migrants increased and no MDA was performed. The sensitivity of PACD was very high. Regarding the timeliness (symptom onset to diagnosis time), it gradually improved from 72 h in 2012 to 12 h in 2017 (83% decrease).

This was a resource intensive strategy with an annual cost of approximately 200,000 euros, that is in line with similar PACD programs [37,38].

Although the PACD program in Evrotas contributed to the reduction of disease transmission in the area after the cluster peak of 2011 due to the implementation of multiple public health interventions, it was not feasible to accurately estimate the impact of each intervention.

Recent literature [27,37,38] and our six-year experience support the need for standardized evaluation indicators and cost effectiveness studies for PACD programs focusing on *P. vivax*, to ensure the most efficient allocation of oftentimes limited public health resources. In addition, the use of novel applications such as mobile information systems and other innovations should be actively explored and assessed, which may significantly decrease the cost and increase accessibility for these populations.

Regarding *P. vivax* infections, further evidence and tools are needed for the diagnosis of subclinical cases, the use and development of sensitive field diagnostic tests, as well as for targeted Mass Drug Administration, especially in view of new eradication drugs (e.g., tafenoquine) or new drug delivery methods [18,27,38,45].

## 5. Conclusions

The geographical area of Evrotas, southern Greece, is an area receptive and vulnerable to malaria transmission, due to vector abundance combined with coexistence of migrants from *P. vivax* endemic countries, heightening the parasite importations in the area.

Although the PACD program was resource intensive, its continuous implementation each season contributed significantly to the timely diagnosis and appropriate radical treatment of each malaria case. Despite the high and continuous influx of newly arrived migrants, along with the other interventions to eliminate locally acquired cases in the area, the PACD program was effective. Moreover, the same results (zero malaria cases) were sustained even after the end of the MDA intervention in 2015.

Taking into consideration the huge recent refugee and migration crisis, this PACD program could serve as good practice and an effective model for implementation in non-endemic, but receptive, areas that serve as transit or final destinations of large migrant populations, thus reducing the risk of malaria introduction and re-establishment, as is the case in several Mediterranean and southern European countries [2,7].

## Figures and Tables

**Table 1:** Characteristics of the population covered by malaria Pro-Active Case Detection program, Evrotas Municipality, 2012–2017.

Characteristics		2012		2013		2014		2015		2016		2017	
Number (%) of new migrants registered, per country of origin	Pakistan	1258	(92%)	547	(90%)	287	(71%)	426	(99%)	1086	(99%)	1138	(99%)
	Afghanistan	61	(4.4%)	5	(0.81%)	68	(17%)	6	(1.4%)	7	(0.63%)	3	(0.26%)
	Bangladesh	36	(2.6%)	58	(9.4%)	39	(10%)			3	(0.27%)	3	(0.26%)
	Other ^1^	6	(0.58%)	1	(0.16%)	8	(2%)			3	(0.27%)	8	(0.69%)
	**Total**	**1361**	(100%)	**611**	(100%)	**402**	(100%)	**432**	(100%)	**1099**	(100%)	**1152**	(100%)
Sex	Male	1361	(100%)	611	(100%)	402	(100%)	432	(100%)	1099	(100%)	1151	(99.9%)
Median (range) age, years		25	(10–65)	26	(14–63)	26	(15–63)	23	(9–53)	23	(10–58)	22	(14–59)
Median (range) number of years of education		8	(0–16)	8	0–16	8	(0–16)	8	(0–16)	8	(0–16)	9	(0–16)
Median time (range) from arrival in Greece to registration in Evrotas, days		720	(1–7920)	1080	(7–10,800)	1440	(6–10,440)	30	(1–5400)	180	(1–8640)	120	(1–6120)
Median time (range) from arrival in Evrotas municipality to registration, days		90	(1–6120)	60	(1–5040)	10	(1–1620)	5	(1–540)	15	(1–540)	7	(1–270)
Malaria history		67/1361	(4.9%)	22/611	(3.6%)	13/402	(3.2%)	21/432	(4.9%)	31/1099	(2.8%)	30/1152	(2.6%)
Documentation status in Greece		99/1361	(7%)	79/611	(13%)	176/402	(45%)	40/432	(9.2%)	66/1099	(6%)	35/1152	(3%)

^1^ Other: India, Iran, Nepal, Nigeria, Rwanda.

**Table 2:** Number of migrants screened, fever screenings and diagnostic tests performed per year, malaria Pro-Active Active Case Detection program, Evrotas Municipality, 2012–2017.

Characteristics	2012	2013	2014	2015	2016	2017
Median number (range) of migrants screened (during weekly or bimonthly visits)	920 (800–1110)	582 (602–859)	496 (445–663)	384 (359–453)	857 (733–1017)	934 (856–1107)
Number of fever screenings	13,833	14,322	13,767	2762	16,535	22,950
Number of Rapid Diagnostic Tests (RDTs) performed ^1^	622	241	196	169	546	514
Number of blood smears for malaria performed ^1^	760	186	160	133	292	205
Number of PCR tests for malaria performed ^1^	369	3	4	87	57	36

^1^ In 2012, 344 RDTs, 439 blood smears and 266 PCRs were performed in the framework of 27 Re-Active Case Detections (focus investigation).

**Table 3:** Number of reported malaria cases ^1^ by epidemiological case classification, Evrotas Municipality, 2012–2017.

Epidemiological Case Classification	2012	2013 ^2^	2014 ^2^	2015	2016	2017
Imported	17	0	0	7	15	14
Locally acquired	10	0	0	1	0	0

^1^ All cases were diagnosed as *P. vivax*. ^2^ In 2013 and 2014, tMDA with anti-malarials for *P. vivax* was administered to migrants from endemic countries residing in Evrotas.

**Table 4:** Number of malaria cases among migrants detected through Pro-Active Case Detection program, Municipality of Evrotas, 2012–2017.

2012	2015	2016	2017
n/N (%)	n/N (%)	n/N (%)	n/N (%)
15/17 (88%)	6/6 (100%)	12/14 (86%)	14/14 (100%)

n = Number of malaria cases in migrants detected through the Pro-Active Case Detection (PACD) program; N = Total number of reported malaria cases in migrants during the months of operation of the PACD program.

**Table 5:** Characteristics and incidence of imported malaria cases, Evrotas Municipality, 2012–2017.

Characteristics	2012 (*n* = 17)		2015 (*n* = 7)		2016 (*n* = 15)		2017 (*n* = 14)	
	Number (%)		Number (%)		Number (%)		Number (%)	
Male	17	(100%)	7	(100%)	15	(100%)	14	(100%)
Country of origin: Pakistan	13	(77%)	7	(100%)	14	(93%)	14	(100%)
Malaria history	4	(24%)	1	(17%)	4	(27%)	5	(36%)
Fever on diagnosis (T ≥ 37.5 °C)	7	(41%)	3	(43%)	5	(33%)	6	(43%)
Low grade fever on diagnosis (T 37 °C–37.4 °C)	2	(12%)	2	(28%)	7	(47%)	4	(28%)
Normal temperature on diagnosis (T < 37 °C)	8	(47%)	2	(29%)	3	(20%)	4	(29%)
	Median (Range)		Median (Range)		Median (Range)		Median (Range)	
Temperature (°C)	37.4 (36–39.9)		37.4 (36.7–40)		37.2 (36.2–42)		37.3 (36.4–38.7)	
Age (years)	23 (10–40)		24 (19–30)		20 (16–40)		19 (15–30)	
Time from arrival in Greece to symptom onset, days	210 (20–1500)		30 (6–90)		165 (60–330)		120 (36–225)	
Incidence (%) ^1^	1.8%		1.8%		1.8%		1.5%	

^1^ Median population was used as the denominator of the incidence.

**Table 6:** Indicators of malaria Pro-Active Case Detection program for malaria cases actively detected (n), Municipality of Evrotas, 2012–2017.

Indicators of ACD	2012 (*n* = 15)	2015 (*n* = 6)	2016 (*n* = 12)	2017 (*n* = 14)
Median time from first symptom (fever) to first contact with the health system	3 days	2.5 days	1.5 days	0.5 days
Median time from first symptom (fever) to diagnosis	3 days	2.5 days	2 days	0.5 days
Median time from first contact with the health system to testing	0 days	0 days	0 days	0 days
Median time from diagnosis to treatment initiation	0 days	0 days	0 days	0 days

**Table 7:** Breslow tests (overall and pairwise comparisons) per year regarding timeliness.

**Pairwise Comparisons**	**Year**	**2012**	**2015**	**2016**
	**2015**	0.674		
	**2016**	0.594	0.972	
	**2017**	0.017	0.234	0.033
**Overall Comparisons**	0.062			

## References

[1] World Health Organization (2019). World Malaria Report 2019.

[2] World Health Organization (2015). Global Technical Strategy for Malaria 2016–2030.

[3] Odolini S., Gautret P., Parola P. (2012). Epidemiology of Imported Malaria in the Mediterranean Region. Mediterr. J. Hematol. Infect. Dis..

[4] Stark K., Schöneberg I. (2012). Increase in malaria cases imported from Pakistan to Germany in 2012. Eurosurveillance.

[5] Odolini S., Gautret P., Kain K., Smith K., Leder K., Jensenius M., Coyle C.M., Castelli F., Matteelli A. (2014). Imported Plasmodium vivax Malaria ex Pakistan. J. Travel Med..

[6] European Centre for Disease Prevention and Control (2019). Malaria Annual Epidemiological Report for 2017.

[7] European Centre for Disease Prevention and Control (2017). Rapid Risk Assessment. Multiple Reports of Locally-Acquired Malaria Infections in the EU.

[8] Vakali A., Patsoula E., Spanakos G., Danis K., Vassalou E., Tegos N., Economopoulou A., Baka A., Pavli A., Koutis C. (2012). Malaria in Greece, 1975 to 2010. Eurosurveillance.

[9] Andriopoulos P., Economopoulou A., Spanakos G., Assimakopoulos G., Andriopoulos P. (2013). A local outbreak of autochthonous Plasmodium vivax malaria in Laconia, Greece—A re-emerging infection in the southern borders of Europe?. Int. J. Infect. Dis..

[10] National Public Health Organization of Greece Epidemiological Surveillance Report: Malaria in Greece 2012. https://eody.gov.gr/wp-content/uploads/2019/01/Malaria_Report_2012_FINAL_23_82013_EN.pdf.

[11] National Public Health Organization of Greece Epidemiological Surveillance Report: Malaria in Greece 2014. https://eody.gov.gr/wp-content/uploads/2019/01/Malaria_annual_report_2014_EN_final.pdf.

[12] Danis K., Baka A., Lenglet A., Van Bortel W., Terzaki I., Tseroni M., Detsis M., Papanikolaou E., Balaska A., Gewehr S. (2011). Autochthonous Plasmodium vivax malaria in Greece, 2011. Eurosurveillance.

[13] European Center for Disease Prevention and Control/World Health Organization Regional Office for Europe (2012). Mission Report.

[14] World Health Organization (2007). WHO Malaria Elimination: A Field Manual for Low and Moderate Endemic Countries.

[15] World Health Organization (2012). Disease Surveillance for Malaria Elimination: An Operational Manual.

[16] National Public Health Organization of Greece Epidemiological Surveillance Report: Malaria in Greece 2013. https://eody.gov.gr/wp-content/uploads/2019/01/Malaria_annual_report_2013_June_2014.pdf.

[17] National Public Health Organization of Greece Epidemiological Surveillance Report: Malaria in Greece 2015. https://eody.gov.gr/wp-content/uploads/2019/01/Malaria_report_26_08_2015.pdf.

[18] Tseroni M., Baka A., Kapizioni C., Snounou G., Tsiodras S., Charvalakou M., Georgitsou M., Panoutsakou M., Psinaki I., Tsoromokou M. (2015). Prevention of Malaria Resurgence in Greece through the Association of Mass Drug Administration (MDA) to Immigrants from Malaria-Endemic Regions and Standard Control Measures. PLoS Negl. Trop. Dis..

[19] Integrated Surveillance and Control Programme for West Nile Virus and Malaria in Greece (MALWEST Project). http://www.malwest.gr/.

[20] Linton Y.M., Smith L., Koliopoulos G., Zounos A.K., Samanidou-Vogiadjoglou A., Patsoula E. (2007). The Anopheles (Anopheles) maculipenis complex (Diptera: Culicidae) in Greece. J. Nat. Hist..

[21] Danis K., Lenglet A., Tseroni M., Baka A., Tsiodras S., Bonovas S., Tsiodras S. (2013). Malaria in Greece: Historical and current reflections on a re-emerging vector borne disease. Travel Med. Infect. Dis..

[22] Hellenic Statistical Authority Table. The De Jure (Registered) Population 2011. https://www.statistics.gr/en/2011-census-pop-hous.

[23] World Health Organization (2016). WHO Malaria Terminology.

[24] World Health Organization (2016). WHO Informal Consultation on Fever Management in Peripheral Health Care Settings: A Global Review of Evidence and Practice.

[25] World Health Organization Malaria Rapid Diagnostic Performance, Summary Results of WHO Malaria RDT Product Testing: Rounds 1–3 (2008–2011). http://www.wpro.who.int/malaria/NR/rdonlyres/005E574B-FFCD-484C-BEDE-C580324AC2CF/0/RDTMalariaRd3_Summary_FINAL11oct.pdf.

[26] Tseroni M., Pervanidou D., Tserkezou P., Rachiotis G., Pinaka O., Baka A., Georgakopoulou T., Vakali A., Dionysopoulou M., Terzaki I. (2015). Field Application of SD Bioline Malaria Ag Pf/Pan Rapid Diagnostic Test for Malaria in Greece. PLoS ONE.

[27] Cotter C., Sturrock H.J., Hsiang M.S., Liu J., A Phillips A., Hwang J., Gueye C.S., Fullman N., Gosling R.D., Feachem R.G. (2013). The changing epidemiology of malaria elimination: New strategies for new challenges. Lancet.

[28] Velasco E., Gomez-Barroso D., Varela C., Diaz O., Cano R. (2017). Non-imported malaria in non-endemic countries: A review of cases in Spain. Malar. J..

[29] A Khattak A., Venkatesan M., Nadeem M.F., Satti H.S., Yaqoob A., Strauss K.A., Khatoon L., Malik S.A., Plowe C.V. (2013). Prevalence and distribution of human Plasmodium infection in Pakistan. Malar. J..

[30] Naeem M.A., Ahmed S., Khan S.A. (2018). Detection of asymptomatic carriers of malaria in Kohat district of Pakistan. Malar. J..

[31] Amirshekari M.B., Nateghpour M., Raeisi A., Haghi A.M., Farivar L., Edrissian G. (2016). Determination of Asymptomatic Malaria among Afghani and Pakistani Immigrants and Native Population in South of Kerman Province, Iran. Iran. J. Parasitol..

[32] Starzengruber P., Fuehrer H.-P., Ley B., Thriemer K., Swoboda P., Habler V.E., Jung M., Graninger W., Khan W.A., Haque R. (2014). High prevalence of asymptomatic malaria in south-eastern Bangladesh. Malar. J..

[33] Harris I., Sharrock W.W., Bain L.M., Gray K.-A., Bobogare A., Boaz L., Lilley K., Krause D., Vallely A.J., Johnson M.-L. (2010). A large proportion of asymptomatic Plasmodium infections with low and sub-microscopic parasite densities in the low transmission setting of Temotu Province, Solomon Islands: Challenges for malaria diagnostics in an elimination setting. Malar. J..

[34] Waltmann A., Darcy A.W., Harris I., Koepfli C., Lodo J., Vahi V., Piziki D., Shanks G.D., Barry A.E., Whittaker M. (2015). High Rates of Asymptomatic, Sub-microscopic Plasmodium vivax Infection and Disappearing Plasmodium falciparum Malaria in an Area of Low Transmission in Solomon Islands. PLoS Negl. Trop. Dis..

[35] Longley R.J., França C., White M., Kumpitak C., Sa-Angchai P., Gruszczyk J., Hostetler J.B., Yadava A., King C.L., Fairhurst R. (2017). Asymptomatic Plasmodium vivax infections induce robust IgG responses to multiple blood-stage proteins in a low-transmission region of western Thailand. Malar. J..

[36] Piperaki E.-T., Tseroni M., Kallimani A., Mavrouli M., Tsakris A., Georgitsou M., Routsias J., Veneti L., Georgakopoulou T., Chania M. (2015). Assessment of Antibody Responses in Local and Immigrant Residents of Areas with Autochthonous Malaria Transmission in Greece. Am. J. Trop. Med. Hyg..

[37] Sutcliffe C.G., Kobayashi T., Hamapumbu H., Shields T., Mharakurwa S., Thuma P.E., Louis T.A., Glass G., Moss W.J. (2012). Reduced Risk of Malaria Parasitemia Following Household Screening and Treatment: A Cross-Sectional and Longitudinal Cohort Study. PLoS ONE.

[38] Sturrock H.J.W., Hsiang M.S., Cohen J., Smith D.L., Greenhouse B., Bousema T., Gosling R.D. (2013). Targeting Asymptomatic Malaria Infections: Active Surveillance in Control and Elimination. PLoS Med..

[39] Frontex Annual Risk Analysis 2014 (p. 35). http://frontex.europa.eu/assets/Publications/Risk_Analysis/Annual_Risk_Analysis_2014.pdf.

[40] Hellenic Police Statistics on Illegal Immigration 2018. http://www.astynomia.gr/index.php?option=ozo_content&perform=view&id=78538&Itemid=73&lang=.

[41] Evlampidou I., Danis K., Lenglet A., Tseroni M., Theocharopoulos Y., Panagiotopoulos T. (2015). Malaria knowledge, attitudes and practices among migrants from malaria-endemic countries in Evrotas, Laconia, Greece, 2013. Eurosurveillance.

[42] Spanakos G., Alifrangis M., Schousboe M.L., Patsoula E., Tegos N., Hansson H., Bygbjerg I., Vakalis N.C., Tseroni M., Kremastinou J. (2013). Genotyping Plasmodium vivax isolates from the 2011 outbreak in Greece. Malar. J..

[43] Spanakos G., Snounou G., Pervanidou D., Alifrangis M., Rosanas-Urgell A., Baka A., Tseroni M., Vakali A., Vassalou E., Patsoula E. (2018). Genetic Spatiotemporal Anatomy of Plasmodium vivax Malaria Episodes in Greece, 2009–2013. Emerg. Infect. Dis..

[44] Sonden K., Castro E., Trönnberg L., Stenstrom C., Tegnell A., Farnert A. (2014). High incidence of Plasmodium vivax malaria in newly arrived Eritrean refugees in Sweden since May 2014. Eurosurveillance.

[45] Lacerda M.V., Llanos-Cuentas A., Krudsood S., Lon C., Saunders D.L., Mohammed R., Yilma D., Pereira D., Espino F.E., Mia R.Z. (2019). Single-Dose Tafenoquine to Prevent Relapse ofPlasmodium vivaxMalaria. N. Engl. J. Med..

